# Self-awareness of falls and its influencing factors in older patients with cardiometabolic multimorbidity: a latent profile analysis

**DOI:** 10.3389/fpubh.2026.1827109

**Published:** 2026-07-06

**Authors:** Zheyuan Xia, Xiang Wang, Leran Tang, Ting Yao, Qiao Hu, Xiao Wang, Yukuan Miao

**Affiliations:** 1 The School of Nursing, Anhui University of Chinese Medicine, Hefei, China; 2 Laboratory of Geriatric Nursing and Health, Anhui University of Chinese Medicine, Hefei, China; 3 Emergency Intensive Care Unit (EICU), The First Affiliated Hospital of Anhui Medical University, Hefei, China

**Keywords:** cardiometabolic multimorbidity, latent profile analysis, nursing care, older adults, self-awareness of falls

## Abstract

**Background:**

Self-awareness of falls is a comprehensive psychological trait encompassing an individual’s subjective assessment of their own falls risk, risk identification, and prospective preventive behavioral tendencies. It serves as a key cognitive-behavioral mediating factor influencing the occurrence of falls. Older patients with cardiometabolic multimorbidity face multiple fall-related risks due to advanced age, multimorbidity, and polypharmacy. However, research on self-awareness of falls in this population remains in its early stages, and most existing studies have adopted a variable-centered approach, which fails to capture the potential individual heterogeneity.

**Methods:**

Using convenience sampling, a total of 363 older patients with cardiometabolic multimorbidity were recruited from six communities in Anhui Province, China, between September and December 2025. Demographic and disease-related information were collected using a general information questionnaire. Assessments were conducted using the following instruments: the Self-awareness of Falls Scale, the STEADI Stay Independent Brochure Questionnaire, the Barthel Index, the Self-Rating Anxiety Scale (SAS), the Multidimensional Scale of Perceived Social Support, and the Pittsburgh Sleep Quality Index. Latent profile analysis was performed using Mplus 8.7 to identify distinct profiles of self-awareness of falls. Univariate analysis and multivariate logistic regression were conducted using SPSS 27.0 to explore factors associated with latent profile membership.

**Results:**

A total of 343 valid questionnaires were collected in this study, yielding an effective response rate of 94.5%. Based on the characteristics of self-awareness of falls in older patients with cardiometabolic multimorbidity, latent profile analysis identified four distinct latent profiles: Low Falls Alertness Profile (Profile 1, *n* = 82, 23.91%), External Environment-Oriented Falls Alertness Profile (Profile 2, *n* = 105, 30.61%), Self-Behavior-Oriented Falls Alertness Profile (Profile 3, *n* = 95, 27.70%), and High Falls Alertness Profile (Profile 4, *n* = 61, 17.78%). The entropy value was 0.856, indicating good accuracy of the latent profile classification. Multivariate logistic regression analysis revealed that multiple factors were significantly associated with latent profile membership of self-awareness of falls in older patients with cardiometabolic multimorbidity. Specifically, aged 65–74 years and having two chronic diseases were strong positive predictors of the Low Falls Alertness Profile and the Self-Behavior-Oriented Falls Alertness Profile (OR = 3.67–4.18, all *p* < 0.05). Male gender was an independent positive predictor of the External Environment-Oriented Falls Alertness Profile (OR = 3.109, *p* < 0.05). Self-rated falls risk score exhibited consistent protective effects across all profiles (OR = 0.323–0.840, all *p* < 0.05).

**Conclusion:**

The level of self-awareness of falls among older patients with cardiometabolic multimorbidity remains at a moderate level. Latent profile analysis identified four distinct subtypes of self-awareness of falls in this population, exhibiting significant heterogeneity. Based on the characteristics of each profile and the differences in influencing factors, clinical nursing practice should move beyond traditional uniform interventions and implement profile-specific assessment and targeted interventions according to different falls alertness subtypes, thereby enhancing the effectiveness of falls prevention.

## 1 Introduction

Falls constitute the leading cause of injury, disability, and even mortality among older adults, with incidence rates rising sharply with advancing age (1, 2). Beyond falls, multimorbidity also poses a pronounced health threat to the aging population. Among these, Cardiometabolic Multimorbidity (CMM), one of the most prevalent patterns of multimorbidity in older adults, is defined as the coexistence of two or more cardiometabolic diseases (including hypertension, diabetes mellitus, dyslipidemia, heart disease, and stroke) (3). CMM is characterized by stable disease clustering and a high prevalence rate (4, 5).

Older patients with CMM, as a high-risk group doubly burdened by frailty and multimorbidity, have a significantly higher falls risk than the general older population or those with a single disease. The underlying mechanisms primarily involve pathophysiological changes induced by CMM, such as hemodynamic instability, vestibular dysfunction, and impaired balance (6, 7), as well as side effects from individual medications or drug–drug interactions, including dizziness, hypoglycemia, and hypotension (8). Crucially, these physiological alterations not only directly compromise physical balance but may also indirectly diminish an individual’s ability to perceive and judge falls risk by interfering with cognitive function. Consequently, older adults with CMM constitute a priority population for falls prevention and control.

The World Falls Guideline Task Force, in its published guidelines for falls prevention among older adults (9), emphasizes that effective falls prevention begins with an individual’s accurate perception of their own falls risk, a perceptive capacity that can be quantitatively assessed through the concept of self-awareness of falls. This construct was first introduced by Shyu et al. (10) and is currently defined in the academic literature as a comprehensive psychological trait encompassing an individual’s perception and judgment of existing or potential falls risks, as well as their corresponding intention to engage in preventive behaviors (11). As a modifiable cognitive psychological factor, maintaining an appropriate level of self-awareness of falls enables individuals to proactively adopt protective behaviors, thereby significantly reducing the risk of falls (12). However, for older patients with CMM, who experience substantial physiological fluctuations and complex pathological conditions, the formation and maintenance mechanisms of self-awareness of falls may be considerably more intricate.

Currently, in terms of research methodology, existing studies on self-awareness of falls have predominantly employed a variable-centered regression analysis paradigm (13, 14). Such studies treat self-awareness of falls as a homogeneous continuous variable within the population, revealing only the average associations between self-awareness of falls and related factors while failing to capture individual heterogeneity. Consequently, when applied to the complex population of older patients with CMM, this approach cannot adequately address the heterogeneity in risk characteristics nor meet the needs for differentiated interventions. Regarding research perspective, most studies have focused on the impact of negative psychological states such as fear of falling on falls occurrence (15), with insufficient attention given to self-awareness of falls as a more prospective and modifiable psychological trait. Furthermore, the study populations have largely been confined to healthy older adults or patients with a single disease (16), and there remains a lack of research that incorporates CMM specific factors to conduct in-depth analyses of the latent profile characteristics of self-awareness of falls in this particular population.

Latent Profile Analysis (LPA) is a person centered statistical classification method that can precisely identify homogeneous subgroups, thereby providing an ideal tool for elucidating the heterogeneity of self-awareness of falls among older patients with CMM. This study focused on older patients with CMM and aimed to identify the latent profiles of self-awareness of falls using LPA, as well as to examine the multidimensional factors influencing profile membership, ultimately providing an empirical basis for the development of targeted falls prevention strategies.

## 2 Subjects and methods

### 2.1 Study design and participants

Using convenience sampling, a total of 363 older patients with CMM were recruited from six communities in Anhui Province, China, between September and December 2025.

The inclusion criteria were as follows: ① aged 65 years or older; ② diagnosed with CMM, defined as the coexistence of at least two cardiometabolic diseases (including hypertension, diabetes mellitus, dyslipidemia, heart disease, and stroke) (3, 17). Specifically, hypertension was diagnosed based on systolic blood pressure ≥140 mmHg (1 mmHg = 0.133 kPa) and/or diastolic blood pressure ≥90 mmHg, or a confirmed diagnosis of hypertension, or use of antihypertensive medication within the preceding two weeks (18). Diabetes mellitus was diagnosed based on a confirmed diagnosis of diabetes or receipt of glucose lowering treatment (oral hypoglycemic agents or insulin injection) within the preceding two weeks (19). Dyslipidemia was diagnosed based on a confirmed diagnosis of dyslipidemia, use of lipid lowering medication within the preceding two weeks, or meeting any of the following criteria: low density lipoprotein cholesterol ≥4.1 mmol/L, triglycerides ≥2.3 mmol/L, total cholesterol ≥6.2 mmol/L, and/or high density lipoprotein cholesterol <1.0 mmol/L, or a documented history of treatment (20). Stroke was diagnosed based on a medical history of stroke and/or transient ischemic attack (TIA) or receipt of any related treatment (21). Heart disease was diagnosed based on a medical history of myocardial infarction, coronary heart disease, angina pectoris, congestive heart failure, or other cardiac conditions, or receipt of any related treatment (3). ③ Diagnosis duration of at least six months; ④ conscious and oriented, with visual or auditory abilities sufficient for normal reading and communication.

The exclusion criteria were as follows: ① severely limited in activities of daily living; ② concurrent vital organ failure or malignant tumor. Cognitive dysfunction was rapidly assessed using the Mini-Cog Assessment Scale (Mini-Cog) (22): after trained researchers explained the assessment requirements, the assessment was conducted through two tasks: “3-word recall” and “clock drawing.” The total score of Mini-Cog = 3-word recall score (0–3 points) + clock drawing score (0–1 point; a correct clock drawing means being able to draw a closed circle, with correct number positions and hands pointing to the specified time). Participants with a total score ≤ 2 points were determined to have cognitive dysfunction and were excluded. ③ severe cognitive impairment or history of psychiatric illness; ④ current participation in other interventional studies.

The sample size was calculated using the formula for estimating a single population proportion: *n* = Z^2^ * *p* * (1-p)/e^2^. Based on previous literature, the prevalence of CMM among individuals aged over 65 years in China was assumed to be 27.7%. With a 95% confidence level (*α* = 0.05) and a 5% margin of error, the calculated minimum sample size was 308 participants. After accounting for an anticipated 15% non response rate, the final sample size was expanded to 363 participants.

### 2.2 Study procedure

The research team consisted of seven members, including three coordinators, two quality control officers, and two data managers. All team members received standardized training prior to the investigation, covering the inclusion and exclusion criteria, standardized instructions, communication skills, questionnaire completion procedures, and data entry protocols.

A face-to-face questionnaire survey was conducted by trained investigators at community health service centers or patients’ homes. The investigators provided full-process guidance on questionnaire completion on-site, promptly corrected or supplemented questionable or missing items, and ensured that all questionnaires were fully completed without missing values. The time required to complete each questionnaire was kept between 20 and 30 min. Prior to the survey, investigators fully explained the research purpose, implementation procedures, and confidentiality principles to each participant, and obtained written informed consent from all participants after they clearly understood the study details. For participants with a primary school education or below who were unable to read independently, the investigator read the entire informed consent form aloud to ensure they fully grasped the relevant requirements. In such cases, participants provided fingerprint signatures instead of written signatures in the presence of an independent witness unaffiliated with the research team; both the witness and the investigator cosigned the form to confirm the completeness and validity of the consent process.

Investigators maintained a neutral attitude while assisting participants in completing the questionnaires. All questionnaires were collected on site, and preliminary verification was conducted within 24 h, with any ambiguous items or missing data clarified promptly. Questionnaires were excluded if the response rate was below 90%, completion time was less than 15 min, or if non differentiated responses were identified. Data that passed the initial review were independently entered by two researchers and subsequently cross checked to ensure accuracy.

### 2.3 Variables and instruments

Regarding the selection of influencing factors, potential determinants of self-awareness of falls in older patients with CMM were identified through a literature review based on the conceptual framework of self-awareness of falls, followed by discussion within the research team. Sociodemographic characteristics (7 items) and disease related factors (3 items) were integrated into a general information questionnaire. Six additional variables, namely self-awareness of falls, self-rated falls risk, activities of daily living, anxiety level, perceived social support, and sleep quality, were measured using validated instruments.

#### 2.3.1 General information questionnaire

Data were collected using a self designed general information questionnaire, which comprised the following two sections. Sociodemographic characteristics included seven variables: gender, age, educational level, marital status, use of assistive devices when walking, presence of a sedentary lifestyle (defined as daytime sedentary time exceeding 6 h per day), and presence of family caregivers. Disease related information included three variables: number of chronic diseases (2 types, ≥3 types), number of medications or injectable treatments received (0, 1 types, ≥2 types), and history of falls within the past year.

#### 2.3.2 Self-awareness of falls scale (SAFE)

The Self-awareness of Falls Scale, developed by Shyu et al. (10), is used to measure older adults’ alertness regarding falls prevention. The scale comprises 21 items across four dimensions: Awareness of Activity Safety and Environment (8 items), Awareness of Physical Functions (6 items), Awareness of Medication (3 items), and Awareness of Cognitive Behavior (4 items). Each item is rated on a 5 point Likert scale, with items 1, 4, 6, 8, and 15 requiring reverse scoring. The total score ranges from 21 to 105, with scores exceeding 54 indicating a relatively high level of self-awareness of falls (23). In this study, the Cronbach’s *α* coefficient for the scale was 0.818.

#### 2.3.3 STEADI stay independent brochure questionnaire (SIB)

The Stay Independent Brochure Questionnaire was developed in 2011 by the Veterans Affairs Greater Los Angeles Healthcare System (VAGLAHS) and its affiliated institutions (24) to assess older adults’ self perceived level of falls risk. The questionnaire consists of 12 items, each answered with “yes” or “no.” For the first two items, a “yes” response is scored as 2 points; for the remaining 10 items, a “yes” response is scored as 1 point. All “no” responses are scored as 0 points. The total score ranges from 0 to 14, with a score of 4 or higher indicating the presence of falls risk, and higher scores reflecting greater risk. According to the 2019 version of the STEADI falls risk assessment criteria (25), if an older adult’s self rated score is below 4, the investigator must inquire whether the individual has experienced a falls within the past year. A history of falls in the past year classifies the older adult as being at risk for falls. In this study, the Cronbach’s *α* coefficient for the questionnaire was 0.788.

#### 2.3.4 Barthel Index (BI)

The Barthel Index was designed by Mahoney and Barthel in the mid-1950s (26) to assess an individual’s capacity to perform activities of daily living. This scale consists of 10 items (the 10 item Barthel Index), including dressing, bathing, grooming, feeding, bowel control, bladder control, bed to chair transfer, toilet use, ambulation on level surfaces, and stair climbing. Each item is rated into four levels based on the need for and extent of assistance required. The total score ranges from 0 to 100, with 100 indicating complete independence, 60 to 99 indicating mild dependence, 41 to 59 indicating moderate dependence, and 0 to 40 indicating severe dependence or complete dependence. In this study, the Cronbach’s *α* coefficient for the scale was 0.861.

#### 2.3.5 Self-rating anxiety scale (SAS)

The Self-Rating Anxiety Scale was developed by Zung (27) to assess the severity of anxiety symptoms as subjectively perceived by individuals. The scale consists of 20 items rated on a 4 point Likert scale, with scores ranging from 1 to 4 representing “none or a little of the time” to “most or all of the time.” The total score is calculated by summing the scores of all items and multiplying by 1.25, with higher scores indicating greater levels of anxiety. A total score below 50 is considered normal, 50 to 60 indicates mild anxiety, 61 to 70 indicates moderate anxiety, and scores above 70 indicate severe anxiety. In this study, the Cronbach’s *α* coefficient for the scale was 0.843.

#### 2.3.6 Multidimensional scale of perceived social support (MSPSS)

The Multidimensional Scale of Perceived Social Support was developed by Zimet et al. (28) to assess an individual’s self perceived social support. The scale comprises three dimensions with 12 items: family support (4 items), friend support (4 items), and significant other support (4 items). Items are rated on a 7 point Likert scale ranging from “very strongly disagree” to “very strongly agree,” corresponding to scores of 1 to 7. The total score ranges from 12 to 84, with higher scores indicating greater levels of perceived social support. Total scores of 12 to 36 represent low social support, 37 to 60 represent moderate social support, and 61 to 84 represent high social support. In this study, the Cronbach’s *α* coefficient for the scale was 0.811.

#### 2.3.7 Pittsburgh sleep quality index (PSQI)

The Pittsburgh Sleep Quality Index was developed by Buysse et al. (29) to assess sleep quality among adults over the preceding month. The scale consists of 19 self rated items and 5 observer rated items, with the 19th self rated item and the 5th observer rated item not contributing to the total score. The 18 self rated items included in scoring are categorized into seven components: subjective sleep quality, sleep latency, sleep duration, habitual sleep efficiency, sleep disturbances, use of sleep medication, and daytime dysfunction. Items are rated on a 4 point Likert scale ranging from 0 (no difficulty) to 3 (very difficult). The total score ranges from 0 to 21, with higher scores indicating poorer sleep quality, and a score greater than 5 suggesting the presence of sleep disturbances. In this study, the Cronbach’s *α* coefficient for the scale was 0.836.

### 2.4 Statistical analysis

SPSS 27.0 software was used for statistical analysis. For continuous data, normality was first evaluated using the Shapiro–Wilk test, and homogeneity of variance was assessed via the Levene test. Continuous data that followed a normal distribution and had homogeneous variance were expressed as mean ± standard deviation, and one-way analysis of variance (ANOVA) was used for comparisons among multiple groups; if the data did not follow a normal distribution or the variance was heterogeneous, they were expressed as median (interquartile range), and the Kruskal-Wallis H test was adopted for inter-group comparisons. Categorical data were presented as frequency and percentage (rate), and the chi-square test or Fisher’s exact test was used for inter-group comparisons. For multiple comparisons in univariate analysis, the Bonferroni correction was applied to control Type I error, to avoid false positive results caused by multiple tests.

Differences in general information across latent profile categories were compared using chi square tests or one way analysis of variance as appropriate. Variables demonstrating statistical significance in the univariate analyses were entered as independent variables, with the four latent profiles of self-awareness of falls among older patients with CMM serving as the dependent variable, in a multinomial logistic regression analysis to identify factors influencing profile membership. The significance level was set at *α* = 0.05.

### 2.5 Ethical considerations

This study was carried out in strict compliance with the ethical standards of the institution and was approved by the Ethics Committee of Anhui University of Chinese Medicine (approval no.: AHUCM-HSS-2025008). All participants were fully informed of the study’s objectives and procedures before providing written informed consent voluntarily. The survey was conducted anonymously, and no clinical intervention measures were involved throughout the research process. To ensure the confidentiality of participants’ information, each questionnaire was marked with a unique identification code, and all electronic data were stored on encrypted, password-protected servers.

## 3 Results

### 3.1 General characteristics of older patients with CMM

A total of 363 questionnaires were distributed, of which 343 valid questionnaires were returned, yielding a valid response rate of 94.5%. Among the excluded invalid questionnaires, 12 were due to missing key variable data (e.g., incomplete completion of the falls alertness scale dimensions), 5 were due to excessively short completion time (<10 min, which was less than one-third of the average completion time, indicating careless responding), and 3 were due to obvious logical errors or patterned responding (e.g., consecutive identical options).

Among the study participants, 111 (32.4%) were aged 65 to 74 years, 168 (49.0%) were aged 75 to 84 years, and 64 (18.7%) were aged 85 years and above. There were 180 female participants (52.5%) and 163 male participants (47.5%). Regarding the number of cardiometabolic conditions, 144 participants (42.0%) had two conditions, while 199 participants (58.0%) had three or more conditions. A total of 116 participants (33.8%) were not taking any oral or injectable medications, 108 participants (31.5%) were taking one medication, and 119 participants (34.7%) were taking two or more medications. Detailed demographic and clinical characteristics, along with the results of univariate analyses, are presented in Table 1.

**Table 1:** Univariate analysis of respondents’ general information and potential categories of self-awareness of falls (*n* = 343).

Variables		Overall (*n* = 343)	Low fall alertness profile (*n* = 82)	Self-behavior-oriented fall alertness profile (*n* = 105)	External environment-oriented fall alertness profile (*n* = 95)	High fall alertness profile (*n* = 61)	F/X^2^	*p* value
Gender	Male	163 (47.5)	52 (63.4)	33 (34.7)	56 (53.3)	22 (36.1)	19.164	*p* < 0.001
	Female	180 (52.5)	30 (36.6)	62 (65.3)	49 (46.7)	39 (63.9)		
Age (year)	65–74	111 (32.4)	20 (24.4)	21 (22.1)	34 (32.4)	35 (57.4)	37.316	*p* < 0.001
	75–84	168 (49.0)	37 (45.1)	49 (51.6)	61 (58.1)	18 (29.5)		
	≥85	64 (18.7)	25 (30.5)	25 (26.3)	10 (9.5)	8 (13.1)		
Education	High school or below	169 (49.3)	33 (40.2)	50 (47.6)	55 (57.9)	31 (50.8)	7.67	0.053
	College or above	174 (50.7)	49 (59.8)	55 (52.4)	40 (42.1)	30 (49.2)		
Marital status	Married	256 (74.6)	67 (81.7)	67 (70.5)	75 (71.4)	47 (77.0)	3.772	0.287
	Unmarried/divorced/widowed	87 (25.4)	15 (18.3)	28 (29.5)	30 (28.6)	14 (23.0)		
Use of assistive devices	Yes	174 (50.7)	47 (57.3)	44 (46.3)	53 (50.5)	30 (49.2)	2.225	0.527
	No	169 (49.3)	35 (42.7)	51 (53.7)	52 (49.5)	31 (50.8)		
Sedentary lifestyle^a^	Yes	157 (45.8)	43 (52.4)	22 (23.2%)	79 (75.2)	13 (21.3)	72.475	*p* < 0.001
	No	186 (54.2)	39 (47.6)	73 (76.8)	26 (24.8)	48 (78.7)		
Having caregivers	Yes	226 (65.9)	52 (63.4)	70 (66.7)	61 (64.2)	43 (70.5)	3.172	0.367
	No	117 (34.1)	30 (36.6)	35 (33.3)	34 (35.8)	18 (29.5)		
Number of oral medications/injectable treatments	0	116 (33.8)	29 (35.4)	31 (32.6)	39 (37.1)	17 (27.9)	1.901	0.929
	1 types	108 (31.5)	24 (29.3)	30 (31.6)	33 (31.4)	21 (34.4)		
	≥2 types	119 (34.7)	29 (35.4)	34 (35.8)	33 (31.4)	23 (37.7)		
Number of chronic diseases	2 types	144 (42.0)	62 (75.6)	33 (34.7)	39 (37.1)	10 (16.4)	57.525	*p* < 0.001
	≥3 types	199 (58.0)	20 (24.4)	62 (65.3)	66 (62.9)	51 (83.6)		
Fall history in the past year	Yes	54	9	15	16	14	4.049	0.256
	No	279	73	90	79	47		
Self-rated fall risk (SIB) (score)		6.65 ± 4.15	1.99 ± 2.36	6.36 ± 3.34	8.56 ± 3.17	10.08 ± 2.73	109.352	*p* < 0.001
Barthel Index (score)		83.76 ± 8.02	78.91 ± 7.57	82.19 ± 6.07	86.67 ± 8.42	87.72 ± 6.49	25.126	*p* < 0.001
Anxiety (SAS) (score)		52.60 ± 15.00	39.32 ± 11.76	47.82 ± 12.11	61.13 ± 11.57	63.2 ± 10.73	76.757	*p* < 0.001
Social support (MSPSS) (score)		59.37 ± 13.41	52.15 ± 12.48	66.83 ± 11.40	58.45 ± 12.64	59.07 ± 13.16	21.071	*p* < 0.001
Sleep quality (PSQI) (score)		13.05 ± 4.92	10.94 ± 4.22	9.43 ± 4.05	16.40 ± 3.49	15.75 ± 3.61	72.637	*p* < 0.001

a Daytime sedentary time exceeding 6 h was considered indicative of a sedentary lifestyle.

### 3.2 Results of latent profile analysis of self-awareness of falls in older patients with CMM

The results showed that the scores of self-awareness of falls among the 343 older patients with CMM ranged from 31 to 100, with a mean score of 64.20 ± 13.34. A person-centered latent profile analysis was conducted based on the scores of the four dimensions of self-awareness of falls, and latent profile models ranging from one to five classes were constructed. The model fit indices are presented in Table 2.

**Table 2:** Model fitting results of potential profile analysis model for self-awareness of falls among older patients with CMM (*n* = 343).

Model	AIC	BIC	aBIC	Entropy	*p* value		Class probability (%)
					LMRT	BLRT	
1-Profle	2311.126	4525.659	2316.450	—	—	—	—
2-Profle	1906.646	4124.536	1915.258	0.949	*p* < 0.001	*p* < 0.001	74.1/25.9
3-Profle	1661.072	3941.753	1673.051	0.868	*p* < 0.001	*p* < 0.001	33.0/47.0/20.1
4-Profle	1430.870	3846.697	1446.176	0.856	*p* < 0.001	*p* < 0.001	17.6/28.0/24.4/30.0
5-Profle	1387.163	3856.22	1405.797	0.821	0.1883	*p* < 0.001	9.2/24.4/27.4/21.3/17.6

aBIC, sample size-adjusted Bayesian information criterion; LMR, Lo–Mendell–Rubin adjusted likelihood ratio test. AIC, Akaike information criterion; BIC, Bayesian information criterion; BLRT, bootstrap likelihood ratio test. “*p* < 0.05: The n-category model is significantly superior to the n-l-category model.

Regarding the information criteria, AIC, BIC, and aBIC exhibited an overall decreasing trend as the number of classes increased, with the decline stabilizing after four classes. Although the AIC (1387.163) and aBIC (1405.797) values for the five class model were slightly lower than those for the four class model (AIC = 1430.870, aBIC = 1446.176), the BIC value for the five class model (3856.22) was higher than that for the four class model (3846.697). This suggests that adding a fifth class resulted in limited improvement in model fit while substantially increasing model complexity, indicating a potential risk of overfitting.

In terms of classification accuracy, the entropy values for the two to five class models were all above 0.8, indicating good classification quality. The two class model exhibited the highest entropy value (0.949), followed by the three class model (0.868) and the four class model (0.856), with the five class model showing the lowest entropy value (0.821). Although the entropy value of the four class model was lower than those of the two class and three class models, it still maintained a relatively high level of classification accuracy and was notably higher than that of the five class model. These findings suggest that the four class model not only ensures clarity in sample classification but also effectively captures the heterogeneity across different classes.

The likelihood ratio test results indicated that both the Lo Mendell Rubin likelihood ratio test (LMRT) and the bootstrapped likelihood ratio test (BLRT) supported the adequacy of the two to four class models. For the two class, three class, and four class models, the *p* values for both LMRT and BLRT were less than 0.001, suggesting that the improvement in model fit relative to the respective previous class models was statistically significant. However, for the five class model, the LMRT *p* value was 0.1883 (*p* > 0.05), indicating no statistically significant difference between the five class and four class models, and that the addition of a fifth class did not further optimize model fit.

Regarding class distribution, the smallest class in the four class model accounted for 17.6% of the sample, with no extremely small classes (<5%) present. The class distribution was balanced and held practical clinical interpretability. In contrast, the class proportions in the five class model were more dispersed, ranging from 9.2 to 27.4%, with the smallest class comprising only 9.2%. This dispersion may lead to overlapping class characteristics and increase the difficulty of subsequent behavioral pattern interpretation.

Based on a comprehensive evaluation of model fit indices, the four class model demonstrated the optimal performance in terms of goodness of fit, classification accuracy, and clinical interpretability. Therefore, it was selected as the final model.

### 3.3 Characteristics and naming of latent profiles of self-awareness of falls among older patients with CMM

**Figure 1:**
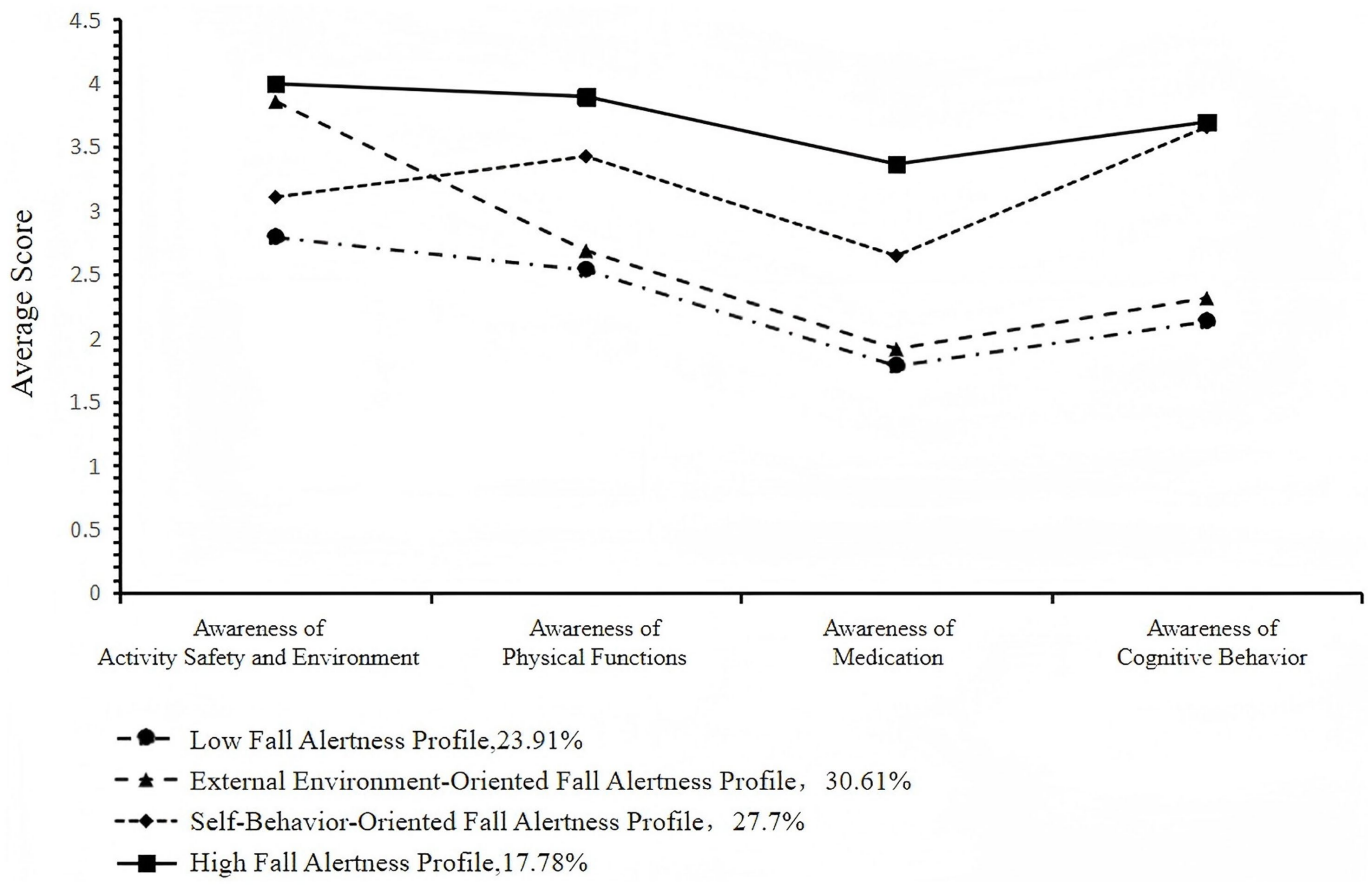
The characteristic distribution of 4 potential profiles of self-awareness of falls among older patients with cardiometabolic multimorbidity.

Based on the latent profile plot of self-awareness of falls among older patients with cardiometabolic multimorbidity (CMM) (Figure 1, “The characteristic distribution of 4 potential profiles of self-awareness of falls among older patients with cardiometabolic multimorbidity”), the characteristics of the four latent classes were analyzed, and each class was named according to its distinctive features.

Profile 1, comprising 82 patients (23.91%), had a mean total score of 52.02 ± 9.51 for self-awareness of falls. This profile exhibited the lowest total score among the four classes, with the lowest scores across all four dimensions: Awareness of Activity Safety and Environment, Awareness of Physical Functions, Awareness of Medication, and Awareness of Cognitive Behavior. Patients in this profile demonstrated a general lack of basic falls risk recognition and proactive prevention awareness. Therefore, this profile was named the Low Falls Alertness Profile.

Profile 2, comprising 95 patients (27.70%), had a mean total score of 67.77 ± 9.33 for self-awareness of falls, ranking second among the four classes. Patients in this profile scored relatively high on the Awareness of Physical Functions, Awareness of Medication, and Awareness of Cognitive Behavior dimensions (all ranking second), but scored low on the Awareness of Activity Safety and Environment dimension (ranking third). This pattern suggests that these patients pay considerable attention to their own physical functioning and demonstrate strong self-perception of falls risk and behavioral prevention, yet exhibit insufficient awareness of external environmental safety and lack vigilance regarding environmental falls risk factors. Therefore, this profile was named the Self-Behavior-Oriented Falls Alertness Profile.

Profile 3, comprising 105 patients (30.61%), had a mean total score of 60.89 ± 10.72 for self-awareness of fall, ranking third among the four classes. Regarding dimensional scores, patients in this profile scored relatively high on the Awareness of Activity Safety and Environment dimension (ranking second) but scored low on the Awareness of Physical Functions, Awareness of Medication, and Awareness of Cognitive Behavior dimensions (ranking third). This pattern suggests that these patients are capable of actively observing and avoiding potential falls risks in their external environment, yet tend to overlook the association between changes in their own health status and falls risk, and demonstrate insufficient vigilance regarding medication induced falls. Therefore, this profile was named the External Environment-Oriented Falls Alertness Profile.

Profile 4, comprising 61 patients (17.78%), had a mean total score of 80.75 ± 8.45 for self-awareness of falls, ranking first among the four classes. Patients in this profile exhibited the highest scores across all four dimensions: Awareness of Activity Safety and Environment, Awareness of Physical Functions, Awareness of Medication, and Awareness of Cognitive Behavior. This profile demonstrated comprehensive and proactive awareness of falls risk prevention. Therefore, this profile was named the High Falls Alertness Profile.

### 3.4 Univariate analysis of latent profiles of self-awareness of falls in older patients with CMM

Univariate analysis revealed statistically significant differences among the four profile groups in terms of gender, age, sedentary lifestyle, number of chronic diseases, self-rated falls risk, Barthel Index score, anxiety level, perceived social support level, and sleep quality (all *p* < 0.05), as presented in Table 1.

### 3.5 Multivariate analysis of latent profiles of self-awareness of falls in older patients with CMM

With self-rated falls risk, Barthel Index score, anxiety level, perceived social support level, and sleep quality scores entered as covariates, and gender, age, sedentary lifestyle, and number of chronic diseases entered as categorical variables (factors), a multinomial logistic regression analysis was conducted with the four latent profiles of self-awareness of falls among older patients with CMM as the dependent variable, using the High Falls Alertness Profile as the reference group. The assignment of variables is presented in Table 3.

**Table 3:** Variable assignment.

Variable	Variable assignment
Gender	1 = male, 2 = female
Age (years)	1 = 65–74, 2 = 75–84, 3 = ≥85
sedentary lifestyle	Yes = 1, No = 2
Number of chronic diseases	1 = 2 types, 2 = ≥3 types
Self-rated fall risk	Actual data
Barthel index	Actual data
Anxiety level	Actual data
Perceived social support	Actual data
Sleep quality	Actual data

As shown in Table 4, with the High Falls Alertness Profile serving as the reference group, multiple factors demonstrated significant associations with distinct latent profiles of self-awareness of falls among older patients with CMM.

**Table 4:** Multifactor analysis of potential profiles of self-awareness of falls among older patients with CMM (*n* = 343).

Variables	Low fall alertness profile (vs. high fall alertness profile)					External environment-oriented fall alertness profile (vs. high fall alertness profile)					Self-behavior-oriented fall alertness profile (vs. high fall alertness profile)				
	β	Odds ratio	95%CI		*p* value	β	Odds ratio	95%CI		*p* value	β	Odds ratio	95%CI		*p* value
			Lower	Upper				Lower	Upper				Lower	Upper	
Gender															
Male	1.62	5.06	0.83	30.97	0.08	1.13	3.11	1.23	7.89	0.02	0.50	1.65	0.49	5.62	0.42
Age (years)															
65–74	1.30	3.67	1.13	11.89	0.03	−0.07	0.93	0.24	3.65	0.92	1.40	4.06	1.25	13.15	0.02
75–84	−1.40	0.25	0.02	2.84	0.26	0.77	2.16	0.56	8.33	0.26	−1.02	0.36	0.07	1.92	0.23
Sedentary lifestyle	−1.73	0.18	0.02	1.40	0.1	−2.61	0.07	0.03	0.19	<0.001	−0.05	0.95	0.26	3.49	0.94
Number of chronic diseases															
2 types	1.43	4.18	1.45	12.06	0.008	−0.99	0.37	0.13	1.07	0.07	−0.44	0.65	0.16	2.66	0.55
Self-rated fall risk	−1.13	0.32	0.22	0.49	<0.001	−0.18	0.84	0.72	0.98	0.02	−0.49	0.61	0.49	0.76	<0.001
Barthel Index	−0.36	0.70	0.61	0.80	<0.001	−0.03	0.97	0.91	1.03	0.29	−0.17	0.84	0.77	0.93	<0.001
Anxiety level	−0.20	0.82	0.75	0.90	<0.001	−0.04	0.97	0.92	1.01	0.12	−0.10	0.90	0.85	0.96	0.001
Perceived social support	−0.15	0.86	0.79	0.94	0.001	−0.02	0.98	0.95	1.01	0.24	0.03	1.03	0.99	1.08	0.19
Sleep quality	−0.22	0.81	0.63	1.04	0.099	0.09	1.09	0.96	1.26	0.18	−0.37	0.69	0.58	0.83	<0.001

For the Low Falls Alertness Profile, significant positive predictors included age 65–74 years (OR = 3.67, 95% CI: 1.13–11.89, *p* = 0.03) and having two chronic diseases (OR = 4.18, 95% CI: 1.45–12.06, *p* = 0.008). Significant negative predictors included self-rated falls risk score (OR = 0.32, 95% CI: 0.22–0.47, *p* < 0.001), Barthel Index score (OR = 0.70, 95% CI: 0.61–0.80, *p* < 0.001), Self-Rating Anxiety Scale score (OR = 0.82, 95% CI: 0.75–0.90, *p* < 0.001), and perceived social support level (OR = 0.86, 95% CI: 0.79–0.94, *p* = 0.001).

For the External Environment-Oriented Falls Alertness Profile, a significant positive predictor was male gender (OR = 3.11, 95% CI: 1.23–7.89, *p* = 0.02), indicating that male patients had a significantly higher probability of belonging to this profile compared to female patients. Significant negative predictors included having a sedentary lifestyle (OR = 0.07, 95% CI: 0.03–0.19, *p* < 0.001) and self-rated falls risk score (OR = 0.84, 95% CI: 0.72–0.98, *p* = 0.02).

For the Self-Behavior-Oriented Falls Alertness Profile, a significant positive predictor was age 65–74 years (OR = 4.06, 95% CI: 1.25–13.15, *p* = 0.02). Significant negative predictors included self-rated falls risk score (OR = 0.61, 95% CI: 0.49–0.76, *p* < 0.001), Barthel Index score (OR = 0.84, 95% CI: 0.77–0.93, *p* < 0.001), Self-Rating Anxiety Scale score (OR = 0.90, 95% CI: 0.85–0.96, *p* = 0.001), and Pittsburgh Sleep Quality Index score (OR = 0.69, 95% CI: 0.58–0.83, *p* < 0.001).

## 4 Discussion

### 4.1 Overall level and latent profile characteristics of self-awareness of falls in older patients with CMM

In this study, the total score on the Self-Awareness of Falls Scale among older patients with cardiometabolic multimorbidity (CMM) was 64.20 ± 13.34, which was higher than that reported for community-dwelling older adults (52.94 ± 8.83) (30) and comparable to values observed in hospitalized older patients (62.67 ± 12.34) (31) and older stroke inpatients (62.74 ± 11.52) (32). This suggests that falls alertness in this population is generally at a moderate-to-high level. Nevertheless, 86 patients (25.07%) scored ≤54, falling below the recommended threshold for falls alertness (23).

Among the four dimensions, the medication alertness subscale exhibited the lowest mean item score (2.36 ± 0.58), reflecting inadequate awareness of medication-related falls risks in these patients. In this study, 66.2% of the participants were taking at least one medication. However, patients often attribute drug-related adverse effects such as dizziness and fatigue to their underlying chronic conditions rather than to medication side effects, thus neglecting the link between medication use and falls risk, this accounts for the relatively low score in this dimension. Moreover, long-term medication use may induce tolerance or habituation to adverse reactions, further diminishing sensitivity to potential falls hazards. Additionally, clinical medication guidance focuses primarily on the management of physiological indicators including blood pressure and blood glucose, with insufficient education regarding falls risk prevention, which further constrains the development of adequate medication alertness.

Previous research has indicated that medication use may enhance falls alertness to a certain degree (33); by contrast, the present study found that medication history had no significant effect on falls alertness. This finding reinforces the aforementioned clinical interpretation: patients generally demonstrate low medication alertness, and their medication experience has not been effectively translated into proactive recognition of medication-related falls risks. Accordingly, clinical nursing practice should prioritize medication safety education to help patients establish a clear association between medication use and falls risk.

### 4.2 Differential effects of multidimensional factors on latent profiles of self-awareness of falls

This study revealed that demographic characteristics (age, gender, sedentary lifestyle), psychosocial factors (anxiety level, social support, sleep quality), and disease-related factors (number of chronic conditions, self-perceived falls risk, and activities of daily living) exerted differential effects on the formation of distinct falls alertness profiles. The gradient patterns observed in the odds ratios delineate a trajectory of patients’ falls risk perception, ranging from “blind spots” to “comprehensive alertness”.

#### 4.2.1 Interactive effects of demographic characteristics and psychosocial factors on falls alertness profiles

Males were more likely to be classified into the external environment-oriented falls alertness profile (OR = 3.11, 95% CI: 1.23–7.89, *p* = 0.02). From the perspective of traditional gender roles, men are socially expected to be strong and independent, which may make them reluctant to acknowledge physical decline and more likely to attribute falls risks to external environmental factors. With regard to risk perception, men tend to underestimate their own health risks and exhibit lower sensitivity to intrinsic hazards such as adverse drug reactions. In terms of health awareness, women are more proactive in acquiring health-related information and maintaining higher alertness regarding medication use and physical function, whereas men are relatively inattentive to endogenous risk signals. These findings support the existence of inherent gender differences in falls risk perception (34).

Sedentary lifestyle was a significant negative predictor of membership in the external environment-oriented falls alertness profile (OR = 0.07, 95% CI: 0.03–0.19, *p* < 0.001). From the perspective of behavioral exposure mechanisms, individuals with sedentary lifestyles exhibit relatively restricted activity ranges, leading to substantially reduced needs for monitoring environmental risks and thereby impeding the development of an external environment-oriented alertness pattern. Furthermore, sedentary behavior may act as an implicit falls prevention strategy (30), in which patients intentionally limit their mobility to avoid falls risks, thus naturally redirecting their risk focus inward rather than toward external environmental factors.

Compared to patients aged 85 years and older, those aged 65–74 years were significant positive predictors of membership in both the Low Falls Alertness Profile (OR = 3.67, 95% CI: 1.13–11.89, *p* = 0.03) and the Self-Behavior-Oriented Falls Alertness Profile (OR = 4.06, 95% CI: 1.25–13.15, *p* = 0.02). These findings suggest that age exerts divergent effects on falls alertness. On one hand, patients in this age group generally exhibit relatively better self-care capacity (as also supported by the significant negative association with the Barthel Index observed in this study, indicating that poorer self-care ability predisposes individuals to the High Falls Alertness Profile). The lack of salient risk signals from functional limitations may lead to a cognitive bias of invulnerability to falls (35), thereby increasing the likelihood of belonging to the Low Falls Alertness Profile. On the other hand, some patients in this age group may gradually increase their alertness to behavior-related falls risks as they begin to experience functional decline, consequently raising their probability of being classified into the Self-Behavior-Oriented Falls Alertness Profile. The ultimate developmental trajectory within this critical period may be further modulated by psychosocial resources and sleep quality.

Anxiety level was a significant negative predictor for both the Low Falls Alertness Profile (OR = 0.82, 95% CI: 0.75–0.90) and the Self-Behavior-Oriented Falls Alertness Profile (OR = 0.90, 95% CI: 0.85–0.96). As an adaptive threat warning signal, anxiety enhances individuals’ sensitivity to falls risks and facilitates the shift of falls alertness patterns from biased perceptions to comprehensive monitoring. However, psychological resources are finite. Patients aged 65–74 years who experience concurrent anxiety and sleep disturbances—a significant negative predictor for the Self-Behavior-Oriented Falls Alertness Profile (OR = 0.69, 95% CI: 0.58–0.83, *p* < 0.001)—may exhibit depletion of their limited cognitive resources (36). Consequently, they may fail to develop a stable self-behavior-oriented alertness pattern, potentially remaining at the stage of risk perception or shifting toward the External Environment-Oriented Falls Alertness Profile.

Social support was a significant negative predictor for the Low Falls Alertness Profile (OR = 0.86, 95% CI: 0.79–0.94, *p* = 0.001). As an important external protective resource, external supervision and daily reminders from family members can help eliminate blind spots in patients’ risk perception. Furthermore, by sharing the burden of risk management, social support alleviates psychological stress and conserves psychological resources, thereby enhancing patients’ active monitoring of falls risks and reducing their likelihood of being classified into the Low Falls Alertness Profile (30).

#### 4.2.2 Gradient effects of subjective falls risk perception and objective health status on falls alertness profiles

Self-rated falls risk score was a significant negative predictor across all three non-high alertness profiles (Low Falls Alertness Profile: OR = 0.32, 95% CI: 0.22–0.47; Self-Behavior-Oriented Falls Alertness Profile: OR = 0.61, 95% CI: 0.49–0.76; External Environment-Oriented Falls Alertness Profile: OR = 0.84, 95% CI: 0.72–0.98; all *p* < 0.05). This indicates that stronger subjective perceptions of falls risk are associated with a higher probability of patients being classified into the High Falls Alertness Profile, suggesting that subjective falls risk perception is a key determinant in the development of falls alertness (37). Further inspection of the gradient changes in odds ratios (Low Falls Alertness Profile < Self-Behavior-Oriented Falls Alertness Profile < External Environment-Oriented Falls Alertness Profile) reveals hierarchical variations in risk perception levels across the three subtypes. The Low Falls Alertness Profile reflects a perceptual blind spot regarding falls, with patients exhibiting almost complete absence of risk awareness. The Self-Behavior-Oriented Falls Alertness Profile is characterized by risk perception limited to individually controllable factors, representing a transitional stage from perceptual blind spot to heightened alertness. Although the External Environment-Oriented Falls Alertness Profile displays relatively higher perception levels, its attribution bias toward external environmental factors results in an imbalanced alertness pattern.

Barthel Index score was a significant negative predictor for both the Low Falls Alertness Profile (OR = 0.70, 95% CI: 0.61–0.80) and the Self-Behavior-Oriented Falls Alertness Profile (OR = 0.84, 95% CI: 0.77–0.93; both *p* < 0.001), suggesting that functional limitations, as a direct intrinsic risk factor for falls, may prompt individuals to maintain relatively high levels of falls alertness via negative physical experiences. The gradient change in odds ratios (Low Falls Alertness Profile < Self-Behavior-Oriented Falls Alertness Profile) elucidates the pathway through which functional decline shapes the development of falls alertness. The Low Falls Alertness Profile, characterized by relatively well-preserved self-care ability, is associated with a lack of awareness regarding falls risk (38). The Self-Behavior-Oriented Falls Alertness Profile corresponds to a compensatory stage in response to functional decline, triggering a risk response pattern centered primarily on self-behavioral adjustment. As functional decline progresses further, patients’ focus of fall-related concern expands from their own functional capacity to the external environment, leading to a gradual transition toward the High Falls Alertness Profile. This gradient effect facilitates understanding of the dynamic evolutionary mechanism underlying the progression from risk exposure to alertness formation across varying levels of functional status.

Having two chronic diseases was a significant positive predictor for the Low Falls Alertness Profile (OR = 4.18, 95% CI: 1.45–12.06, *p* = 0.01), indicating that patients with fewer comorbidities were more likely to show insufficient alertness to falls risks. This finding is consistent with the study by Li et al. (39). A potential explanation is that patients with fewer comorbidities experience relatively milder physical symptoms and have not yet developed a heightened awareness of falls risk. Furthermore, given the high prevalence of CMM, patients may mistakenly regard their disease status as a normal part of aging, underestimating the insidious effects of cardiometabolic disorders on balance function and consequently maintaining a state of low falls alertness.

### 4.3 Public health, ageing policy, and nursing implications

Patients in the Low Falls Alertness Profile have the lowest fall risk perception and are the priority for both public health programmes and nursing interventions. Objective fall risk screening (e.g., Morse or Tinetti scales) should be converted into personalised feedback, such as comparing balance performance with age norms or using wearable gait indicators, to help patients recognise links between chronic conditions, functional decline and fall hazards. Adults aged 65 to 74 years independently predicted this profile (OR = 3.67). They should be listed as a key target in local ageing screening projects and receive free community based fall risk examinations. Motivational interviewing and mobile phone risk reminders embedded in primary care can correct their underestimation of personal fall risk.

For patients in the Self-Behavior-Oriented Falls Alertness Profile, the public health focus is improving community wide environmental hazard awareness and promoting ageing friendly home renovations, while nursing interventions centre on remedying patients’ blind spot for environmental fall risks. Structured community led hazard identification training can engage patients and caregivers in regular home safety inspections and targeted household modifications. Local health authorities may offer home adaptation subsidies to reduce environmental fall risks at the population level. Anxiety and poor sleep independently predicted this profile (OR = 0.90 and 0.69, respectively). Community mental health services should integrate psychological counselling and sleep management into chronic disease follow up, helping patients shift from a self behavior focused alertness pattern to comprehensive fall risk cognition.

Regarding the External Environment-Oriented Falls Alertness Profile, public health initiatives should strengthen population wide medication safety promotion for older adults with cardiometabolic diseases, and nursing practice guides individuals to expand risk perception from external surroundings to intrinsic physical and pharmaceutical risks. Standardised symptom diaries help users document adverse drug reactions and physical changes, establishing clear links to fall vulnerability. Primary care institutions can arrange periodic free gait, balance and medication safety education as part of routine chronic disease management. Male sex independently predicted this subtype (OR = 3.11). Population based sex specific strategies should use objective gait and balance test results, not verbal persuasion, to help male patients recognise functional decline and improve internal risk vigilance.

Patients in the High Falls Alertness Profile maintain comprehensive fall prevention cognition and behaviors. They can serve as peer health volunteers in community science popularisation, reducing public health costs. Nursing follow up should include periodic anxiety screening to prevent pathological worry and unnecessary activity restriction.

From a cross-cutting perspective on health equity and surveillance, social support was a protective factor against the low alertness profile (OR = 0.86). Governments should subsidise smart fall sensors and wearable devices for socially isolated older adults living alone. To ensure health equity, low tech alternatives (e.g., volunteer home visits, telephone reminders) must be provided alongside high tech interventions for older adults in rural or low income areas with limited digital access. For public health surveillance, health authorities can incorporate a short form of the Self Awareness of Falls Scale into periodic health surveys, allowing tracking of profile distribution over time and evaluation of campaign impacts. Profile tailored digital reminders (e.g., caution for slow standing within 30 min after medication) can be integrated into chronic disease management platforms (40). Telehealth linking community centres and family doctors enables dynamic monitoring of daily living, emotion and sleep, supporting early intervention. These measures reduce the population fall burden and advance an integrated prevention treatment care healthy ageing service system.

### 4.4 Limitations

Although this study provides empirical evidence regarding the heterogeneity of self-awareness of falls among older patients with CMM, several limitations should be acknowledged. First, the cross sectional design employed in this study, while revealing associations between influencing factors and latent profiles of falls alertness, cannot infer causal relationships among variables or the evolutionary process of behavioral patterns across different subtypes. Second, the sample was limited to six communities in Anhui Province, China, and recruited using convenience sampling. Furthermore, although data collection relied on patient self-report scales with established reliability and validity, potential recall bias may still exist, affecting the representativeness and objectivity of the findings. Third, this study did not incorporate community level environmental and care resource indicators, such as community safety facilities, home modification support, and frequency of family physician visits, making it difficult to comprehensively analyze the moderating effects of external environmental resources on different falls alertness profiles.

## 5 Conclusion

This study identified four heterogeneous profiles of self-awareness of falls among older patients with CMM using latent profile analysis. By systematically examining the differential influencing factors across these profiles, the findings reveal the complex heterogeneity in patients’ patterns of falls alertness. Theoretically, this study extends beyond the traditional variable-centered research framework, deepening the understanding of the intrinsic structure of self-awareness of falls from a population heterogeneity perspective. Future research should further validate the classification through prospective studies and develop precise falls intervention strategies integrating individual, family, and community resources based on profile characteristics.

## 6 Future directions

Future research should be expanded in the following directions. First, longitudinal studies should be conducted to explore the dynamic evolution of latent profiles of self-awareness of falls throughout the disease trajectory, thereby validating their stability and predictive validity. Second, objective measurement indicators, including balance function, gait analysis, and wearable device monitoring, should be integrated to multidimensionally validate the behavioral characteristics of different profiles. Third, systematic level variables such as community health service capacity and family support resources should be incorporated to construct a theoretical model of influencing factors encompassing individual, family, and community levels. Furthermore, leveraging real-time monitoring through wearable devices and AI-based early warning systems can facilitate timely interventions involving family members and community healthcare providers, establishing rapid falls alert and emergency response mechanisms to minimize injury to the greatest extent possible.

## Data Availability

The original contributions presented in the study are included in the article/supplementary material, further inquiries can be directed to the corresponding authors.
